# Respiratory sensory gating measured by respiratory-related evoked potentials in generalized anxiety disorder

**DOI:** 10.3389/fpsyg.2015.00957

**Published:** 2015-07-09

**Authors:** Pei-Ying S. Chan, Chia-Hsiung Cheng, Shih-Chieh Hsu, Chia-Yih Liu, Paul W. Davenport, Andreas von Leupoldt

**Affiliations:** ^1^Department of Occupational Therapy, College of Medicine, Chang Gung University, Taoyuan, Taiwan; ^2^Healthy Ageing Research Center, Chang Gung University, Taoyuan, Taiwan; ^3^Department of Psychiatry, Chang Gung Memorial Hospital, Taoyuan, Taiwan; ^4^Department of Medicine, Chang Gung University, Taoyuan, Taiwan; ^5^Department of Traditional Chinese Medicine, Chang Gung University, Taoyuan, Taiwan; ^6^Department of Physiological Sciences, College of Veterinary Medicine, University of Florida, Gainesville, FL, USA; ^7^Research Group on Health Psychology, University of Leuven, Leuven, Belgium; ^8^Department of Systems Neuroscience, University Medical Center Hamburg-Eppendorf, Hamburg, Germany

**Keywords:** respiratory sensation, respiratory sensory gating, respiratory-related evoked potentials, generalized anxiety disorder

## Abstract

The perception of respiratory sensations plays an important role both in respiratory diseases and in anxiety disorders. However, little is known about the neural processes underlying respiratory sensory perception, especially in patient groups. Therefore, the present study examined whether patients with generalized anxiety disorder (GAD) would demonstrate altered respiratory sensory gating compared to a healthy control group. Respiratory-related evoked potentials (RREP) were measured in a paired inspiratory occlusion paradigm presenting two brief occlusion stimuli (S1 and S2) within one inspiration. The results showed a significantly greater S2/S1 ratio for the N1 component of the RREP in the GAD group compared to the control group. Our findings suggest altered respiratory sensory processing in patients with GAD, which might contribute to altered perception of respiratory sensations in these patients.

## Introduction

The experience of respiratory sensations plays not only an important role in respiratory diseases such as asthma or chronic obstructive pulmonary disease (COPD), but also in anxiety disorders. For example, respiratory sensations such as breathlessness and chest tightness as well as ventilatory changes are diagnostic for anxiety disorders suggesting a close relationship between respiratory sensory processing and anxiety symptoms (American Psychiatric Association, 2013). Previous studies have demonstrated that negative affective states and traits including anxiety are related to over-perception of respiratory sensations irrespective of underlying ventilatory changes (Carr et al., 1994; Zaubler and Katon, 1996; Bogaerts et al., 2005; von Leupoldt and Dahme, 2007; von Leupoldt et al., 2013). However, few of the aforementioned studies used objective measures for respiratory perception, and the neural mechanisms that underlie the interrelationships between anxiety and increased perception of respiratory sensations are poorly understood (von Leupoldt et al., 2013).

The respiratory-related evoked potential (RREP) is a useful non-invasive electrophysiological method for studying the effects of anxiety on respiratory perception and its neural processing (von Leupoldt et al., 2013). With a single inspiratory occlusion paradigm (which is similar to the oddball paradigm used to test auditory sensory gating), a past report in healthy participants found that high anxious individuals on average presented increased RREP peak amplitudes for the long-latency peaks during negative emotional compared to neutral stimulation, which is the opposite pattern found in low anxious individuals (von Leupoldt et al., 2010, 2011). In addition, respiratory sensory gating, similar to other types of neural gating with exteroceptive stimuli such as sound and touch (Adler et al., 1998; Arnfred et al., 2001), was used to investigate central neural mechanisms of filtering repetitive respiratory stimuli within a short time window (Chan and Davenport, 2010a). A paired occlusion stimulation paradigm can be used to examine respiratory sensory gating by eliciting paired RREP waveforms, where the second stimulus (S2) results in a smaller N1 peak amplitude compared to the first stimulus (S1) in healthy individuals (Chan and Davenport, 2008). The N1 peak, primarily originating from the bilateral sensorimotor cortices, is thought to reflect both first and second order processing of respiratory sensory information in the cortex (Chan and Davenport, 2010a; von Leupoldt et al., 2013). A smaller RREP N1 peak S2/S1 ratio is indicative of an enhanced respiratory sensory gating function (i.e., filtering out more redundant sensory information).

There has been a robust amount of literature testing sensory neural gating in psychiatric disorders with exteroceptive, but not interoceptive stimuli (Ludewig et al., 2002; Holstein et al., 2010; Hunter et al., 2011; Markham and Koenig, 2011). These studies found that individuals with diseases including panic disorder and obsessive-compulsive disorder (OCD) had deficits in prepulse inhibition and sensorimotor gating (Ludewig et al., 2002; Kohl et al., 2013). It was reasoned that compromised sensory gating functions are associated with sensory overload or sensory “flooding” into the higher cortex, which may result in over-perception (Adler et al., 1998). Interoceptive respiratory stimuli have rarely been used to examine sensory gating of anxious individuals in previous studies. A recent report using RREPs has found that state anxiety modulated respiratory sensory gating, as demonstrated by a larger N1 peak S2/S1 ratio in higher compared to lower non-clinically anxious individuals (Chan et al., 2012). However, it remains unknown how clinical levels of anxiety, such as present in general anxiety disorder, are related to a similarly altered neural processing of respiratory sensations.

Therefore, the purpose of this study was to examine respiratory sensory gating elicited by a paired inspiratory occlusion paradigm in patients with generalized anxiety disorder (GAD). It was hypothesized that compared to a healthy control group, individuals with GAD would show a reduced respiratory sensory gating function as demonstrated by an elevated N1 peak S2/S1 ratio.

## Materials and Methods

### Participants

This study was approved by the Chang Gung Medical Foundation Institutional Review Board. A group of 20 patients with the diagnosis of GAD were recruited from the psychiatric outpatient clinic in a medical center located in northern Taiwan. All patients were interviewed by a psychiatrist with the mini-international neuropsychiatric interview (MINI), which is a structured diagnostic interview for DSM-IV diagnoses (Sheehan et al., 1998). Co-morbid diagnoses of substance abuse or psychosis served as exclusion criteria. A group of age-matched healthy controls (HCs) was recruited through public advertisements. All participants reportedly had no history of respiratory, cardiovascular, or neurological disease. All participants were required not to take any prescribed medication for at least 12 h before the experiment.

### Experimental Procedure

After signing the informed consent, participants completed a standard pulmonary function test (PFT) with a spirometer (Cardinal Health Inc., Dublin, OH, USA). The PFT was conducted based on the guidelines of the American Thoracic Society and European Respiratory Society (Miller et al., 2005). In addition, participants were administered the Chinese-version questionnaires of the Beck Anxiety Inventory (BAI; Beck and Steer, 1990) and the Beck Depression Inventory-II (BDI-II; Beck et al., 1996).

During the experiment, participants were instructed to sit comfortably in an armed chair while breathing through a mouthpiece with their nose occluded. The mouthpiece was connected to a two-way non-rebreathing valve (Hans Rudolph Inc., Kansas City, MO, USA). The inspiratory port of the non-rebreathing valve was connected to a customized occlusion valve (Hans Rudolph Inc., Kansas City, MO, USA). A solenoid of a trigger box was connected to the occlusion valve and a pressure tank through reinforced tubing (Chan and Davenport, 2010a). The occlusion valve closure was manually controlled by the experimenter in the adjacent room. The participants’ mouth pressure was monitored and recorded at the center of the non-rebreathing valve through a differential pressure transducer which connected to the pneumotachograph amplifier (1110 series, Hans Rudolph Inc., Kansas City, MO, USA) and a PowerLab signal recording unit (ADInstruments Inc., Bella Vista, NSW, Australia). For details of the respiratory apparatus setup, please refer to the review of Chan and Davenport (2010a).

The RREP method was previously described in a few comparable studies (Chan and Davenport, 2010b; Chan et al., 2014). In the current study, the electroencephalography (EEG) was sampled from cortical sites C3 and C4 at 1 kHz with a 40-channel EEG system (NuAmps, Compumedics Neuroscan Inc., Charlotte, NC, USA), bandpass filtered from DC to 50 Hz and referenced to the bilateral mastoids. Individual electrode impedance was set below 5 kΩ. The participants were provided with paired inspiratory occlusions of 150 ms each with a 500 ms inter-stimulus-interval. The paired stimuli were provided at the onset of inspiration randomly every 2–4 breaths. The onset of occlusion was identified as the start of mouth pressure change (Labchart V7, ADInstruments Inc., Bella Vista, NSW, Australia). At least 100 paired occlusions were collected for data analysis in every participant. The trigger box was set up to send parallel markers to the Neuroscan 4.5 recording software (Compumedics Neuroscan Inc., Charlotte, NC, USA). The participants were instructed to keep breathing normally, rather than to stop breathing, during inspiratory occlusions, and to count the number of breaths they felt obstructed during the experiment.

### Data Analyses

For offline analysis, the EEG epoch was defined and extracted from 200 ms before to 1000 ms after the respiratory occlusion, i.e., for S1 and S2 separately. The first 200 ms served as the baseline. The signals were corrected for ocular movement using a built-in algorithm of the analysis software (BrainVision Analyzer 2, Brain Products GmbH., Gilching, Germany) and further filtered between 1 and 50 Hz (12 dB/octave roll-off). The artifacts were defined when greater than 100 and 60 μV, baseline to peak, for the four eye electrodes and all other electrodes, respectively. After the artifacts were extracted from the signals, the corresponding epochs were then averaged for S1 and S2 separately. The RREP N1 peak amplitudes for S1 and S2 were identified in a time window of 85–135 ms after occlusion onset at cortical sites C3 and C4 and the S2/S1 ratios were calculated.

Separate one-way analyses of variance (ANOVA) were performed to test for group differences in pulmonary function, non-respiratory parameters, and S2/S1 ratios. Based on our *a priori* hypothesis, the analyses for the N1 peaks were conducted with a one-tailed test. The critical *p* value was set at <0.05.

## Results

Twenty patients with the diagnosis of GAD and 20 age-matched HC were recruited for this study. Data of two patients and two controls were excluded due to incomplete EEG data, which left the study with 18 patients (10 females and eight males) and 18 controls (nine females and nine males) for final analyses. The demographic data and the pulmonary function results of the two groups of participants are shown in Table 1. There was no statistical difference regarding age and pulmonary function between the two groups. The GAD group showed significantly higher scores than the HC group for the BDI-II [*F*(1,35) = 10.31, *p* < 0.01] and BAI [*F*(1,35) = 21.12, *p* < 0.001]. These scores (in the mid-range of the scales) indicated significantly higher levels of depression as well as anxiety in the GAD patients, respectively.

**TABLE 1:** **Baseline characteristics of study groups (group mean ± SD)**.

**Variables**	**GAD patients**	**Healthy controls**
N	18	18
Age (years)	47 ± 8.9	42 ± 9.3
Gender (female/male)	10/8	9/9
FEV1 (L)	2.7 ± 0.9	2.9 ± 0.6
FEV1 of predicted value (%)	86 ± 9.3	81.6 ± 10.7
BDI-II score	15.9 ± 11.1	6.3 ± 6.1*
BAI score	13 ± 7.6	4.2 ± 3*

FEV1, forced expiratory volume in 1 sec; BDI-II, Beck Depression Inventory-II; BAI, Beck Anxiety Inventory. *Indicates a significant difference between the GAD group and the healthy control group (p < 0.05).

**FIGURE 1:**
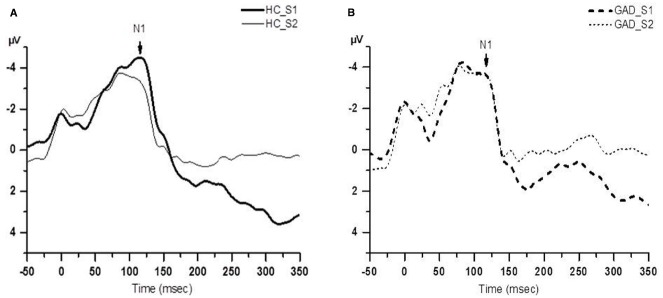
**Group averaged waveform from the C4 electrode. (A)** The black solid lines represents the averaged S1 and S2 waveforms of the healthy control (HC) group (*N* = 18); **(B)** the black dashed lines represents the averaged S1 and S2 waveforms of the GAD group (*N* = 18).

**FIGURE 2:**
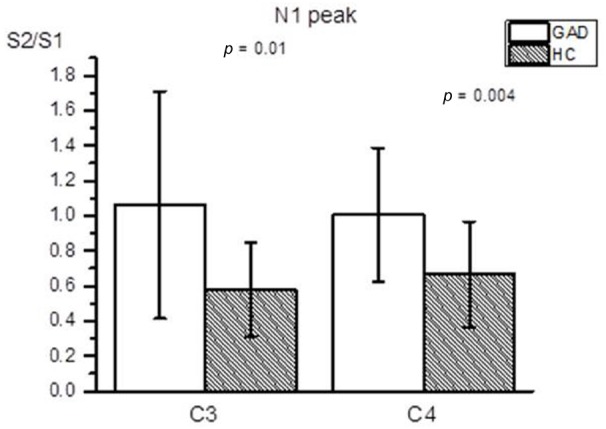
**Averaged RREP N1 peak S2/S1 ratio (mean ± SD) at the C3 and C4 electrodes.** The shaded bars represent the averaged S2/S1 ratios of the HC group. The GAD group showed a significantly greater N1 S2/S1 ratio compared to the healthy controls at the C3 and C4 electrodes (*p* = 0.01 and 0.004, respectively).

Figure 1 shows group averaged S1 and S2 RREP waveforms of the HC group (a) and the GAD group (b). The one-way ANOVA results revealed that the GAD group showed a significantly greater N1 S2/S1 ratio compared to the HCs at both electrode sites [Figure 2; C3: 1.06 ± 0.65 and 0.58 ± 0.27, *F*(1,25) = 6.172, *p* < 0.05; C4: 1.01 ± 0.38 and 0.67 ± 0.3, *F*(1,32) = 8.28, *p* < 0.01]. Further analyses on the S1 and S2 amplitudes for N1 showed that the HC group had significantly greater S1 amplitudes compared to the GAD group at the C3 electrode [*T*(1,27) = 2.505, *p* < 0.05]; however, the independent *t*-test did not show a significant difference for the S1 amplitude at C4 [*T*(1,33) = 0.906, *p* > 0.05]. Similarly, the analyses on the S2 amplitudes at electrodes C3 and C4 did not show significant differences between the two groups [*T*(1,25) = –0.32 and *T*(1,32) = –0.686, respectively, *p* > 0.05].

## Discussion

This study demonstrates that the paired inspiratory occlusion paradigm is a feasible measure for investigating respiratory sensory gating function in GAD patients. The results demonstrate that individuals with GAD, compared to HCs, show a higher RREP N1 S2/S1 ratio, which is suggestive of reduced respiratory sensory gating. This might be related to altered perception of respiratory sensations reported in the patient population.

The present findings are consistent with previous studies in healthy non-clinical samples, in which elevated levels of state anxiety and induced negative emotion were found to reduce respiratory sensory gating as evidenced by RREP methodology (Chan and Davenport, 2010b; Chan et al., 2012; Chenivesse et al., 2014). For example, using a similar paradigm of paired inspiratory occlusions in healthy individuals, Chenivesse et al. (2014) found reduced respiratory sensory gating during unpleasant compared to neutral emotional stimulation using affective picture series. Chan and Davenport (2010b) observed greater S2/S1 ratios for the RREP N1 peak during nicotine-withdrawal induced anxiety in college-aged smokers. In addition, Chan et al. (2012) tested the effect of state anxiety on the RREP in healthy college-aged students and found that higher anxious individuals demonstrated increased N1 S2/S1 ratios compared to lower anxious individuals. The present study suggests that reduced respiratory sensory gating is not only related to mild forms of state or trait anxiety, but might also be evident in more chronic, clinically relevant forms of anxiety such as in GAD. This increased afferent throughput of respiratory sensory signals might lead to an increased awareness of respiratory sensations and contribute to the altered perception of respiratory sensations in patients with anxiety disorders.

In the present study, the group difference in the N1 S2/S1 ratio was related to the fact that the HC group demonstrated higher S1 amplitudes compared to the GAD group for the N1 peak, at least for one out of two analyzed electrode sites. However, in a previous study in healthy participants, it was found that individuals with higher levels of anxiety had higher amplitudes of S2, while no group differences were observed for S1 (Chan et al., 2012). It can be speculated that the discrepancy between the present results and the previous report may be related to the counting task used in the present study, where participants were required to count the number of obstructed breaths during the experiment. Subsequently, the S1 N1 peak amplitude could have been modulated by focused attention generated by counting the stimulus, which needs to be explored further in future systematic studies. However, the observed general effect of the present study demonstrates a significantly smaller amplitude difference between S1 and S2 in GAD patients, which points toward higher sensory throughput. Thus, the present results converge with the findings of Chenivesse et al. (2014), who similarly found a reduction in respiratory sensory gating to be more closely associated with an amplitude modulation of S1 N1 and not S2 N1. The seemingly unaffected S2 amplitude in the present GAD group might be a function of the attentional processes for S1 acting on the neural throughput. However, we cannot fully exclude the alternative explanation that the observed increase in the RREP N1 S2/S1 ratio in the GAD group is unrelated to the concept of central neural gating and merely represent an altered neural response to the first of two paired stimuli in this population. This would be consistent with the notion of altered “gate-in ability” mentioned by Hu et al. (2012) where they found increased auditory S1 amplitude in healthy compared to patients with schizophrenia (Hu et al., 2012). Nevertheless, the extent to which a clinical anxiety disorder impacts respiratory gating at the throughput is unclear and requires further systematic investigation.

The present study is also in line with previous studies using exteroceptive stimuli, which have similarly demonstrated that several anxiety disorders are related to disrupted sensory gating in modalities other than the respiratory modality (Neylan et al., 1999; Ghisolfi et al., 2004; Hashimoto et al., 2008; Stewart and White, 2008; de Leeuw et al., 2010; Nanbu et al., 2010). For example, Hashimoto et al. (2008) tested auditory sensory gating with a paired click paradigm in patients with OCD and found a disrupted P50 S2/S1 ratio. Ghisolfi et al. (2004), using auditory stimulation, reported significantly greater S2/S1 ratios in post-traumatic stress disorder (PTSD) patients, compared to controls. Moreover, Nanbu et al. (2010) found an impaired P50 gating function in the OCD patients represented by elevated S2/S1 ratios with auditory stimuli compared to the HCs. Taken together, these past and the present findings suggest that impaired gating functions in anxiety disorders are not specific for one sensory modality, but can be observed across different interoceptive as well as exteroceptive modalities.

Some cautions need to be exercised when generalizing the results of the present study. Firstly, the study participants only self-reported to have no diagnosis of a respiratory disease or acute respiratory symptoms on the test day, such as experiencing a cold. Secondly, our study did not differentiate between patients with shorter disease duration and those with longer disease duration. Since it is sometimes observed by clinicians that patients with longer disease duration might also report rather reduced levels of respiratory sensations than those with shorter disease duration, future studies are encouraged to examine effect of disease duration on respiratory sensory gating. Finally, we cannot fully rule out residual effects of long-term anxiolytic, antidepressant, or antipsychotic medications in the GAD group. Previous studies have indeed demonstrated the potential modulating effects of medications on sensory gating in patients with anxiety disorders (Siegel et al., 2005; de Leeuw et al., 2010). Although our participants were asked to refrain from medication intake 12 h before the experiments, effects of long-term treatments remain unclear. Future studies are, therefore, encouraged to systematically examine the various potential effects of different treatments on respiratory sensory gating.

In summary, the present study suggests that patients with GAD show a larger S2/S1 ratio for the RREP N1 peak, which is suggestive of reduced respiratory sensory gating. Whether this pattern of neural processing of respiratory information varies between subgroups of GAD patients and the degree of anxiety requires more investigation. Future research is needed to clarify the effects of respiratory symptoms, medications, and duration of the disease on respiratory sensory gating in patients with GAD. Finally, GAD specific responses to the first of two paired respiratory stimuli and the respective impact on the subsequent gating ratio need further investigation.

### Conflict of Interest Statement

The authors declare that the research was conducted in the absence of any commercial or financial relationships that could be construed as a potential conflict of interest.
